# Spontaneous *wbbL* and *waaG* mutations selected by phage vB_EcoP_P61425 confer resistance and attenuate virulence in ExPEC

**DOI:** 10.3389/fmicb.2026.1953743

**Published:** 2026-10-12

**Authors:** Long Chen, Sixiang Xu, Xiaoyue Li, Pei Li, Mengmeng Su, Peifan Liu, Yilin Fang, Wei Zhang

**Affiliations:** 1 Department of Clinical Laboratory, The First People's Hospital of Zhangjiagang City, Zhangjiagang Hospital Affiliated to Soochow University, Zhangjiagang, China; 2 MOE Joint International Research Laboratory of Animal Health and Food Safety, College of Veterinary Medicine, Nanjing Agricultural University, Key Lab of Animal Bacteriology, Ministry of Agriculture, Nanjing, China; 3 College of Life Sciences and Food Engineering, Hebei University of Engineering, Handan, China

**Keywords:** *Escherichia coli*, lipopolysaccharide, phage resistance, phage therapy, virulence trade-offs

## Abstract

Bacteriophage therapy is a promising strategy against multidrug-resistant (MDR) extraintestinal pathogenic *Escherichia coli* (ExPEC), yet bacterial resistance evolution remains a major clinical challenge. In this study, we isolated and comprehensively characterized a novel lytic phage, vB_EcoP_P61425, belonging to the genus *Suseptimavirus*. The phage has a latent period of 15 min and a burst size of approximately 68 PFU/cell. Phage vB_EcoP_P61425 exhibits stability across temperatures of 4 °C to 50 °C and a pH range of 3 to 10, displaying potent lytic activity against common ExPEC serotypes including O25 and O75. Genomic analysis revealed a 77933 bp double-stranded DNA genome with a GC content of 42.60% and 118 predicted coding sequences (CDSs), devoid of known lysogeny-associated, toxin, or antimicrobial resistance genes. *In vitro* assays confirmed its strong bactericidal efficacy, and it significantly reduced bacterial colonization in a medical catheter model. To elucidate the genetic mechanisms underlying bacterial resistance, spontaneous phage-resistant mutants were isolated and analyzed using whole-genome resequencing, targeted gene deletion, and genetic complementation assays. We demonstrated that mutational inactivation or directed deletion of the lipopolysaccharide (LPS) biosynthesis genes *wbbL* and *waaG* significantly impaired phage adsorption, thereby conferring resistance to vB_EcoP_P61425 in EC1649. Crucially, these resistant mutants exhibited phenotypic and virulence-associated trade-offs, manifested by altered cell morphology, markedly impaired adherence to host epithelial cells, and significantly attenuated virulence in a *Galleria mellonella* larval infection model. Collectively, this study demonstrates that mutations in the LPS biosynthesis genes *wbbL* and *waaG* in *E. coli* EC1649 confer phage resistance while imposing an evolutionary trade-off that attenuates bacterial pathogenicity, providing new insights for optimizing phage therapy regimens against ExPEC.

## 1 Introduction

Extraintestinal pathogenic *Escherichia coli* (ExPEC) is a major cause of urinary tract infections (UTIs), bloodstream infections, and neonatal sepsis in humans and animals (Kaper et al., 2004). The global dissemination of multidrug-resistant (MDR) ExPEC, particularly extended-spectrum β-lactamase-producing and carbapenem-resistant strains, has increasingly compromised the efficacy of conventional antibiotics (Boutzoukas et al., 2023; Olaitan et al., 2025). Bacteriophages therefore represent potential alternatives or adjuncts to antibiotics because of their host specificity and bacteriolytic activity, and their clinical use against refractory bacterial infections has produced encouraging results (Aslam et al., 2020; Pirnay et al., 2024). However, the emergence of phage-resistant bacteria remains an important barrier to sustained therapeutic efficacy.

Resistance to phages is frequently mediated by mutations that modify or eliminate bacterial surface receptors, including outer membrane proteins, lipopolysaccharides (LPS), and capsular polysaccharides (Labrie et al., 2010; Gong et al., 2017; Letarov and Kulikov, 2017). Because these structures also contribute to membrane integrity, host colonization, and bacterial virulence, receptor-mediated phage resistance may be accompanied by pleiotropic phenotypic consequences, including reduced adherence or attenuated virulence (León and Bastías, 2015). Identifying the bacterial determinants of phage adsorption and characterizing the phenotypic consequences of resistance are therefore important for evaluating candidate therapeutic phages. Nevertheless, the molecular basis and virulence-associated consequences of phage resistance remain insufficiently characterized in ExPEC.

In this study, we isolated a broad-spectrum lytic phage capable of lysing ExPEC, including APEC and UPEC, and investigated the molecular mechanisms and evolutionary trade-offs associated with bacteriophage resistance. Specifically, we first characterized the biological properties, performed genomic analysis, and evaluated the *in vitro* antibacterial efficacy of the newly isolated *Suseptimavirus* phage vB_EcoP_P61425, while also assessing its ability to inhibit bacterial colonization in a catheter model. Subsequently, to identify the host determinants involved in phage adsorption and bacterial resistance determinants, spontaneous phage-resistant mutants were isolated from EC1649 under phage selection pressure, and the underlying mechanisms were analyzed using whole-genome resequencing, targeted gene knockout, and genetic complementation assays. Finally, the evolutionary trade-offs triggered by phage resistance were investigated by observing bacterial morphology, evaluating the adherence of resistant mutants to human bladder epithelial HTB-9 cells, and assessing their virulence using a *Galleria mellonella* larval infection model. Collectively, this study defines the role of mutations in the LPS biosynthesis genes *wbbL* and *waaG* in conferring resistance to phage vB_EcoP_P61425 in EC1649, providing experimental evidence for exploiting evolutionary and virulence-associated trade-offs to optimize phage therapy strategies against ExPEC.

## 2 Materials and methods

### 2.1 Bacterial strains and growth conditions

Extraintestinal pathogenic *Escherichia coli* (ExPEC) strains were cultured in standard Luria-Bertani (LB) medium. The ExPEC panel included uropathogenic *E. coli* (UPEC) of human origin provided by Zhangjiagang First People's Hospital, avian pathogenic *E. coli* (APEC) isolated from the brain, liver, and heart of chickens originating from Shandong and Jiangsu provinces, China, and sheep pulmonary *E. coli* strains isolated from China. Detailed strain information is listed in Supplementary Table S1, and all these isolates were utilized for phage host range identification.

The UPEC strains of human origin were recovered from residual clinical specimens during routine diagnostic procedures and obtained from the bacterial strain collection maintained by the Department of Clinical Laboratory, Zhangjiagang First People's Hospital. The isolates were fully de-identified before being used in this study. According to the institutional guidelines, formal ethical approval and individual informed consent were not required for this *in vitro* study.

### 2.2 Isolation, purification, and morphological characterization of phage

Raw, untreated medical wastewater samples (*n* = 3) were collected from a hospital in Zhangjiagang, Jiangsu Province, China. The collected samples were transported to the laboratory on ice and processed immediately. Phage isolation and purification were performed using the standard double-layer agar plate technique (Mirza et al., 2025). Sewage samples were centrifuged at 5000 × *g* for 10 min, and the supernatant was filtered through a 0.22 μm sterile filter. A 100 μL aliquot of the filtrate was mixed with 100 μL of EC1649 culture, added to 5 mL of molten 0.5% (w/v) LB semi-solid agar, and poured onto solid LB agar plates. Plates were incubated at 37 °C for 6–8 h, with clear plaque formation indicating phage presence. Single plaques were picked and serially purified for at least five consecutive rounds via double-layer agar assays until uniform plaque morphology was obtained. For phage propagation, purified plaques were inoculated into 5 mL of EC1649 culture, incubated at 37 °C with shaking at 180 rpm for 6 h, and filtered through a 0.22 μm sterile filter to remove host bacteria, yielding a high-titer phage suspension (approximately 10^10^ PFU/mL). The purified phage stock was stored in SM buffer (50 mM Tris-HCl, pH 7.5, 100 mM NaCl, 8 mM MgSO_4_) at 4 °C for routine use and at −80 °C supplemented with 25% (v/v) glycerol for long-term storage.

For morphological characterization, 20 μL of purified phage suspension was dropped onto a copper grid, air-dried, and negatively stained with 2% phosphotungstic acid (PTA) for 30–60 s. Phage morphology was observed and recorded using a Hitachi H-7650 transmission electron microscope (TEM).

The newly isolated phage was designated vB_EcoP_P61425, where “vB” denotes a bacterial virus, “Eco” identifies *E. coli* as the host, “P” denotes a short-tailed morphotype, and “P61425” represents the isolate identifier.

### 2.3 Biological characterization of phage

Thermal stability was evaluated by incubating 1 mL aliquots of phage suspension (2.0 × 10^9^ PFU/mL) at a temperature gradient of 4 °C, 28 °C, 37 °C, 50 °C, 60 °C, 70 °C, and 80 °C for 1 h (Ismael et al., 2024). Phage titers at each temperature were determined via the standard double-layer agar plate method. For pH stability, phages were incubated in SM buffer (pH 1–12) at room temperature for 1 h. Phage titers for all stability assays were determined via the double-layer agar plate method.

Overnight cultures of EC1649 were subcultured (1:100) in fresh LB broth and grown at 37 °C with shaking at 180 rpm until they reached the logarithmic growth phase (OD_600_ = 0.5). The bacterial suspension was adjusted to a concentration of 1.0 × 10^8^ CFU/mL using fresh LB broth and kept on ice during manipulation prior to the assay. Phage vB_EcoP_P61425 was added to 8 mL of EC1649 culture (10^8^ CFU/mL) at multiplicity of infection (MOI) gradients of 10, 1, 0.1, 0.01, 0.001, and 0.0001. The mixtures were incubated at 37 °C with shaking at 180 rpm for 10 h. After incubation, the samples were centrifuged at 5000 × *g* for 5 min, and the supernatants were filtered through a 0.22 μm sterile filter. Phage titers were determined via the double-layer agar plate method. The initial phage-to-bacterium ratio that produced the highest progeny phage titer after 10 h of incubation was defined as the optimal MOI for phage propagation.

For the one-step growth curve, EC1649 at 1.0 × 10^8^ CFU/mL was infected with the phage at an MOI of 0.1 and incubated at 37 °C for 5 min to allow phage adsorption. This relatively low MOI was selected to minimize multiple infection of individual bacterial cells and thereby facilitate reliable determination of the latent period and burst size. The mixture was then centrifuged at 5,000 × *g* for 5 min at 4 °C. The supernatant was discarded, and the pellet was resuspended in 1 mL of LB broth. The suspension was transferred into 8 mL of fresh LB, and timing was initiated immediately. A 100 μL aliquot was collected at 0 min to determine the initial concentration of infected bacterial cells. The culture was incubated at 37 °C with shaking at 180 rpm for 2 h. Phage titers were measured every 2 min for the first 10 min, every 5 min from 10 to 30 min, and every 10 min from 30 to 120 min. The burst size was calculated as the ratio of phage titer at the plateau phase to the initial concentration of infected bacterial cells. All experiments were performed in triplicate.

### 2.4 Host-range determination and efficiency of plating

The host range of phage vB_EcoP_P61425 was initially screened using a spot assay. Briefly, 100 μL of each bacterial culture adjusted to approximately OD_600_ = 0.5 was mixed with 5 mL of molten 0.5% (w/v) LB semi-solid agar and poured onto an LB agar plate. After solidification, 10 μL of the phage suspension at 1.0 × 10^9^ PFU/mL was spotted onto the bacterial lawn. The plates were incubated at 37 °C for 12–16 h.

Isolates producing lysis zones in the spot assay were further examined using the double-layer agar plaque assay. 10-fold serial dilutions of the phage suspension were prepared in SM buffer. Each phage dilution (100 μL) was mixed with 100 μL of bacterial culture and 5 mL of molten 0.5% (w/v) LB semi-solid agar and then poured onto an LB agar plate. After incubation at 37 °C for 12–16 h, plaques were counted to determine the phage titer. Each isolate was tested in triplicate. An isolate was considered susceptible when discrete plaques were reproducibly detected in all three replicate assays.

The relative efficiency of plating (EOP) was calculated as the mean phage titer obtained on each test isolate divided by that obtained on the propagation host, *E. coli* EC1649. EOP values were classified as high (EOP ≥ 0.5), medium (0.1 ≤ EOP < 0.5), low (0.001 < EOP < 0.1), or inefficient (EOP ≤ 0.001).

### 2.5 Phage genome sequencing and bioinformatics analysis

Genomic DNA was extracted from an enriched phage suspension (1.0 × 10^9^ PFU/mL) using the λ Phage Genomic DNA Extraction Kit (Zomanbio, Beijing, China) according to the manufacturer's instructions. The extracted DNA was sequenced by Novogene (Beijing, China) using the Illumina HiSeq platform. The quality of the raw sequencing reads was assessed using FastQC v0.12.1, and adapter sequences and low-quality bases were removed using Trimmomatic v0.40. The resulting clean reads were assembled *de novo* using SPAdes v3.11.1. The complete genome sequence of vB_EcoP_P61425 was deposited in the National Center for Biotechnology Information (NCBI) database with the accession number PZ091942.

CDS were predicted and functionally annotated using Pharokka v1.9.1 with default parameters (Bouras et al., 2022). A linear genome annotation map was generated using the Proksee web server. The genome was screened for antimicrobial-resistance genes, virulence-associated genes, and lysogeny-associated genes using PhageScope with default parameters. Phages related to vB_EcoP_P61425 were identified using BLASTn against the NCBI nucleotide collection database. Only complete phage genomes were retained, and the 20 genomes with the highest BLASTn maximum scores were selected for comparative genomic analysis. Pairwise intergenomic similarities were calculated using VIRIDIC with default parameters (Moraru et al., 2020). A global viral proteomic tree was generated using Viral Proteomic Tree Server (VipTree) based on genome-wide tBLASTx similarities (Nishimura et al., 2017). A local proteomic subtree comprising vB_EcoP_P61425 and 11 related phages was subsequently extracted from the ViPTree output. ViPTree calculates genome-wide similarity scores (SG) from tBLASTx results and constructs the proteomic tree using the BIONJ algorithm based on the corresponding genomic-distance matrix. Comparative genomic synteny between vB_EcoP_P61425 and *Escherichia* phage ECBP2 was visualized using Easyfig v2.2.5. Pairwise sequence similarities were calculated using tBLASTx with default parameters, and homologous regions were shaded according to their sequence identities.

### 2.6 *In vitro* antibacterial activity and catheter colonization assays

Overnight cultures of EC1649 were subcultured (1:100) in fresh LB broth and grown at 37 °C with shaking at 180 rpm until the culture reached the logarithmic growth phase (OD_600_ = 0.5). The bacterial suspension was adjusted to a concentration of 1.0 × 10^8^ CFU/mL using fresh LB broth and kept on ice during manipulation prior to the assay. A 3 mL aliquot of the phage suspension was mixed with 3 mL EC1649 (10^8^ CFU/mL) at MOIs of 10, 1, and 0.1, respectively. The mixture was adjusted to 20 mL with LB broth and incubated at 37 °C with shaking at 180 rpm for 6 h. Untreated bacterial growth controls contained sterile PBS, and blank controls contained sterile LB broth only. OD_600_ was recorded every 10 min for the first 2 h and then every 1 h from 2 to 6 h to generate growth inhibition curves. Each group was assayed in triplicate.

To evaluate the inhibitory effect of phage vB_EcoP_P61425 on biofilm formation, sterile medical catheter segments (2 cm) were inoculated with 100 μL of EC1649 (10^8^ CFU/mL) and incubated at 37 °C for 1 h. Subsequently, 100 μL of phage suspension (10^9^, 10^8^, or 10^7^ PFU/mL) was added to assess the inhibition of biofilm development. Control segments received 100 μL of sterile PBS. After 3, 6, 12, and 24 h of co-incubation, adherent bacteria were detached by vortexing in PBS and quantified by plate counting. All experiments were performed in triplicate.

### 2.7 Isolation and phenotypic characterization of phage-resistant mutants

The isolation of phage-resistant mutants was performed as previously described with minor modifications (Wen et al., 2024). Specifically, 100 μl of phage vB_EcoP_P61425 (10^9^ PFU/mL) and 100 μl of EC1649 (10^8^ CFU/mL) were mixed at a multiplicity of infection (MOI) of 10 in 5 mL of LB medium (0.5% agar). The mixture was poured onto sterile LB solid medium and incubated at 37 °C for 24 h. If single colonies appeared, they were passaged five times on LB medium to ensure genetic inheritance of the mutation. After passaging, different strains were subjected to the double-layer agar plate method, and 3 μl of vB_EcoP_P61425 was applied to the surface, followed by incubation at 37 °C for 6 h. If no plaques appeared or the plaques became turbid, the strain was considered a phage-resistant mutant. To observe the changes in colony morphology of the resistant mutants, EC1649 and the phage-resistant mutants were streaked onto LB solid medium and incubated at 37 °C for 16 h, and the colony morphology was observed.

### 2.8 Whole-genome resequencing and mutation identification

To elucidate the genetic basis of phage resistance, parental strain EC1649 and stable phage-resistant mutants were subjected to whole-genome resequencing. Genomic DNA was extracted from overnight bacterial cultures using the FastPure Bacteria DNA Isolation Mini Kit (Vazyme, Nanjing, China) according to the manufacturer's instructions. DNA concentration was accurately quantified using a Quantus™ Fluorometer (Promega, USA) with Quant-iT™ PicoGreen dsDNA reagent (Thermo Fisher Scientific, USA), and DNA structural integrity was verified by 1.0% agarose gel electrophoresis (5 V/cm, 20 min). All qualified DNA samples exhibited clear intact bands without degradation before library construction. Whole-genome sequencing was performed on the Illumina NovaSeq PE150 platform by Beijing Novogene (Beijing, China) with an average sequencing depth of >100-fold. Raw reads were quality-filtered using FastQC and Trimmomatic to remove adapter sequences and low-quality bases. *De novo* genome assembly of parental strain EC1649 was carried out using SPAdes version 3.11.1 to generate the reference genome. To identify specific mutation sites in the resistant mutants, Breseq version 0.39.0 was used to align the clean sequencing reads to the reference genome (EC1649), identify variations such as SNPs and InDels, and screen and locate key specific mutation sites. Potential candidate genes related to phage resistance were screened, and the mutated genes were functionally annotated by BLASTx.

### 2.9 Construction of gene-deletion and complemented strains

The *wbbL* gene (coding region 1-374 bp) and *waaG* gene (coding region 1-441 bp) in EC1649 were knocked out using the pDM4 suicide plasmid. Briefly, the upstream and downstream homologous arms of *wbbL* and *waaG* were amplified by PCR and fused, and the resulting fragments were ligated into the linearized pDM4 vector using the ClonExpress^®^ II One Step Cloning Kit. The successfully constructed recombinant plasmids pDM4-*wbbL* and pDM4-*waaG* were extracted and transformed into *E. coli* s17-λpir competent cells. Positive clones were screened on LB agar plates supplemented with 50 μg/mL Cm^r^, and sequence accuracy was verified by Sanger sequencing. Conjugal transfer was performed between the recombinant plasmid-carrying s17-λpir strains and the parental strain EC1649, and the transconjugants were selected on LB agar plates containing working concentrations of both 50 μg/mL Amp^r^ and 50 μg/mL Cm^r^. Positive colonies from the dual-resistant plates were inoculated into LB liquid medium supplemented with 15% sucrose for the second homologous recombination event. Finally, successful gene knockout was verified by PCR using specific in/out primers, and the deletion strains EC1649Δ*wbbL* and EC1649Δ*waaG* were obtained.

For the construction of recombinant complemented plasmids pGEN-*wbbL* and pGEN-*waaG*, the full-length coding sequences of *wbbL* and *waaG* were amplified from the genomic DNA of EC1649, respectively. The amplified products were ligated into the linearized pGEN vector using the same homologous recombination method described above. The ligation products were transformed into DH5α via heat shock, and successful transformants were selected on LB agar supplemented with 50 μg/mL Apr^r^, followed by colony PCR verification. The verified recombinant plasmids pGEN-*wbbL* and pGEN-*waaG* were subsequently extracted. Plasmid pGEN-*wbbL* and pGEN-*waaG* was electroporated into the corresponding gene deletion strains constructed above (electroporation parameters: 1.8 kV, 0.1 cm cuvette). Successful plasmid transformation was confirmed by colony PCR and Sanger sequencing. The PCR primer sequences are listed in Supplementary Table S2. The PCR verification results for the deletion mutants are shown in Supplementary Figure S1, and the verification results for the complemented strains are shown in Supplementary Figure S2.

### 2.10 Bacterial colony morphology and TEM examination

EC1649, EC1649Δ*wbbL*, EC1649Δ*waaG* were streaked on TSA supplemented with 5% sheep blood and incubated overnight at 37 °C. Colony morphology was visually assessed. For TEM, exponential-phase cultures were harvested, washed with PBS, fixed in 2.5% glutaraldehyde at 4 °C, and examined using a Hitachi H7650 electron microscope.

### 2.11 Spot test and phage adsorption assay

For the spot test, 10 μL of phage suspension was spotted onto a bacterial lawn (5 mL soft agar containing 100 μL of overnight bacterial culture), and lysis was assessed after overnight incubation at 37 °C. The phage adsorption assay was performed as previously described, with minor modifications (Li et al., 2025). 500 μL of EC1649 (10^8^ CFU/mL) and 500 μL of vB_EcoP_P61425 (10^8^ PFU/mL) were mixed. The mixture was incubated at 37 °C for 10 min. Following incubation, the mixture was centrifuged at 5000 × *g* for 5 min. Then, 100 μL of the supernatant was taken to calculate the phage titer. A non-adsorption control was prepared by mixing 500 μL of LB liquid medium with 500 μL of phage suspension vB_EcoP_P61425. The adsorption rate was calculated as ((PFU_LB_ – PFU_treat_) / PFU_LB_) × 100%. Each experiment was repeated three times.

### 2.12 Human epithelial cell adhesion assay

HTB-9 cells were preserved in our laboratory. The detailed culture procedures were as follows: HTB-9 cells were routinely cultured in RPMI-1640 medium supplemented with 5% bovine serum and 1% penicillin-streptomycin at 37 °C in a humidified atmosphere containing 5% CO_2_. For the adhesion assay, HTB-9 cells were seeded into 24-well cell culture plates at a density of 5.0 × 10^5^ cells/well and incubated for 24 h to form a confluent monolayer. Prior to bacterial infection, cell monolayers were washed three times with sterile PBS and replaced with serum-free and antibiotic-free RPMI-1640 medium to completely eliminate residual antibiotics and prevent any interference with bacterial viability. EC1649, UPR1, UPR2, UPR3, EC1649Δ*wbbL*, EC1649Δ*waaG*, and their complemented strains were cultured to mid-log phase, washed three times with serum-free and antibiotic-free RPMI-1640, and added to wells at 5 × 10^7^ CFU per well. Plates were centrifuged at 800 × *g* for 10 min to promote bacterial-cell contact and incubated at 37 °C (5% CO_2_) for 1 h. Thereafter, cells were washed 5–7 times with sterile PBS to thoroughly remove non-adherent bacteria. To release adherent bacteria, 1 mL of sterile ddH_2_O was added to each well to lyse the cells. Cell lysates were homogenized by pipetting, serially 10-fold diluted in sterile PBS, and plated onto LB agar plates. After incubation at 37 °C for 12–16 h, colonies were enumerated. The absolute adhesion rate was first calculated as: (adherent count / initial inoculum count) × 100%. To facilitate comparison across strains, the relative adhesion rate was subsequently calculated by normalizing the absolute adhesion rate of each mutant or complemented strain against that of the wild-type strain EC1649 (set as 100%): relative adhesion rate = (absolute adhesion rate_sample_ / absolute adhesion rate_EC1649_) × 100%. All assays were performed with three independent biological replicates.

### 2.13 *In vivo* virulence evaluation in the *Galleria mellonella* larval model

Virulence of different strains was evaluated using the *G. mellonella* infection model (Williamson et al., 2014). Nine groups were included: EC1649, UPR1, UPR2, UPR3, EC1649Δ*wbbL*, EC1649Δ*waaG*, EC1649Δ*wbbL*-p*wbbL*, EC1649Δ*waaG*-p*waaG* and PBS control. Healthy *G. mellonella* larvae (~3 cm in length, weighing 250–300 mg) were purchased from Keyun Bio (Henan Jiyuan Baiyun Industry Co., Ltd., Jiyuan, China). Standardized larvae with uniform cream-colored bodies, active motility, and no signs of melanization or dark spots were selected for experiments. Larvae were acclimated at 37 °C for 24 h prior to infection. Each larva was injected with 20 μL of 5 × 10^4^ CFU/mL bacterial suspension through the last pair of prolegs. Larval mortality was monitored every 12 h for 72 h. Larvae were considered dead when they displayed complete melanization and failed to respond to physical tactile stimulation. Each group contained 10 larvae, and all assays were performed with three independent biological replicates. Survival curves were plotted using the Kaplan-Meier method, and statistical differences between groups were evaluated using the Log-rank (Mantel-Cox) test.

### 2.14 Statistical analysis

All experiments were performed with at least three independent biological replicates, and quantitative data are presented as the mean ± standard deviation (SD). Statistical analyses were conducted using GraphPad Prism v8.0.

For the thermal and pH stability assays, differences among experimental conditions were analyzed using one- way ANOVA followed by Dunnett's multiple-comparisons test, with 4 °C and pH 7 as the respective reference conditions. Initial and final phage titers obtained at different MOIs were analyzed using two-way ANOVA followed by Šídák's multiple-comparisons test to compare the initial and final titers at each MOI. The longitudinal bacterial-growth data were analyzed using two-way repeated-measures ANOVA, with treatment and time as factors, followed by Dunnett's multiple-comparisons test comparing each phage-treated group with the untreated bacterial control at each time point. Data from the catheter colonization assay were analyzed using ordinary two-way ANOVA, with treatment and time as factors, followed by Dunnett's multiple-comparisons test comparing each treatment group with the PBS control at each time point. For the phage adsorption and epithelial-cell adhesion assays, comparisons between EC1649 and the spontaneous phage-resistant mutants were performed using one-way ANOVA followed by Dunnett's multiple-comparisons test. Comparisons among EC1649, the gene-deletion strains, and the corresponding complemented strains were performed using one-way ANOVA followed by Tukey's multiple-comparisons test. Survival curves were generated using the Kaplan–Meier method and compared using the log-rank (Mantel–Cox) test. When multiple pairwise comparisons were performed, *P* values were adjusted using the Holm–Šidák method. All tests were two-sided, and *P* < 0.05 was considered statistically significant.

## 3 Results

### 3.1 Results of phage isolation, identification, and biological characterization

TEM revealed that vB_EcoP_P61425 possesses an elongated capsid with a length of 112 ± 0.5 nm and a width of 45 ± 0.5 nm, along with a short tail with multiple tail fibers measuring approximately 17 ± 0.5 nm in length (Figure 1A). The lytic activity of this phage was confirmed using the double-layer agar plaque assay (Figure 1B). The phage remained stable at temperatures from 4 to 50 °C, showed a significant decline in titer at 60 °C, and was completely inactivated at 70 °C (Figure 1C). The phage remained stable across a pH range of 3 to 10 (Figure 1D). An MOI of 10 produced the highest progeny phage titer after 10 h and was therefore identified as the optimal MOI for phage propagation (Figure 1E). The one-step growth curve indicated a 15 min latent period with a plateau at 40 min, and a burst size of approximately 68 PFU/cell (Figure 1F). Spot assays produced lysis zones in 66 of the 123 isolates (53.66%). Among these 66 spot-positive isolates, 39 reproducibly formed discrete plaques in the double-layer agar assay and yielded quantifiable EOP values, corresponding to a plaque-confirmed host range of 31.71% (39/123). The distribution of spot-positive isolates according to sample source is summarized in Table 1, and the detailed spot-assay and EOP results for individual isolates are provided in Supplementary Table S1.

**Figure 1:**
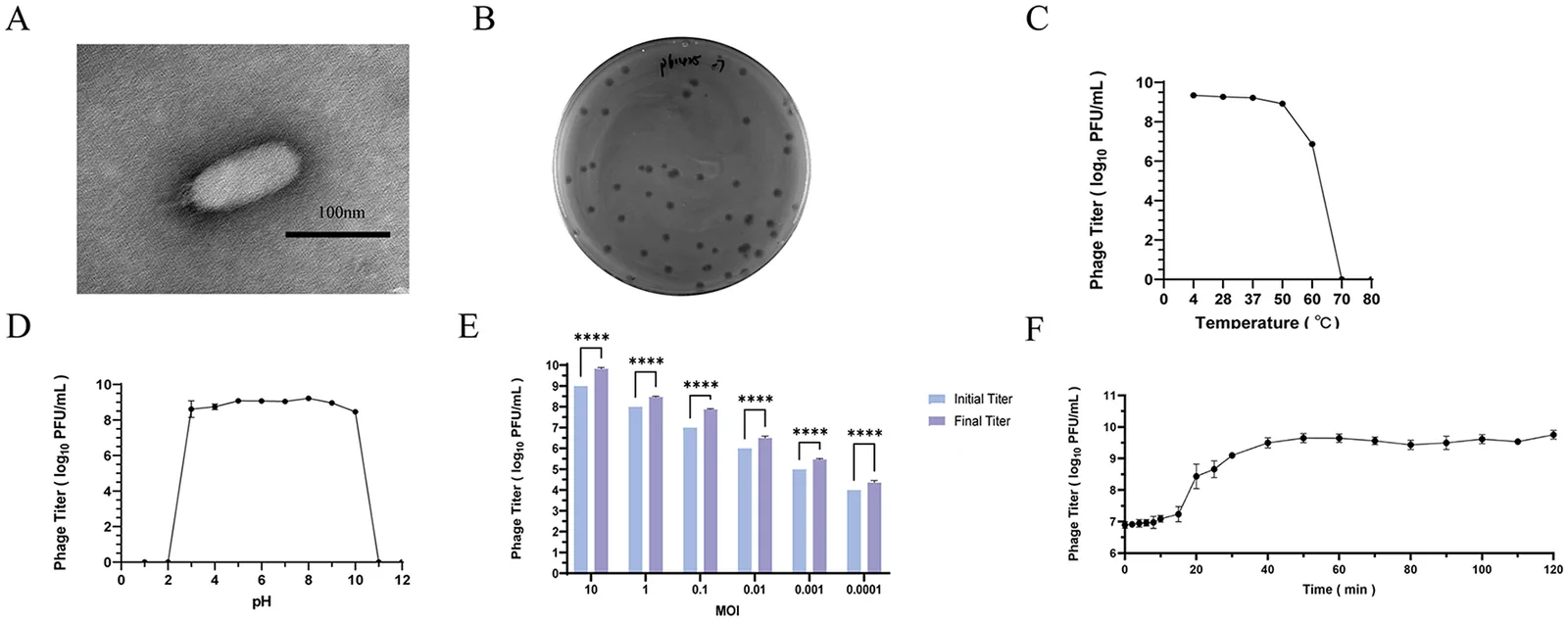
Morphological and biological characterization of phage vB_EcoP_P61425. **(A)** Transmission electron microscopy image of phage vB_EcoP_P61425 (scale bar = 100 nm). **(B)** Plaque morphology of phage vB_EcoP_P61425 formed on host strain EC1649. **(C)** Thermal stability of the phage **(D)** pH stability of the phage **(E)** Phage titers before and after incubation with EC1649 at different multiplicities of infection (MOIs) for 10 h. **(F)** One-step growth curve of vB_EcoP_P61425 in EC1649 at an MOI of 0.1. Data are presented as the mean ± SD of three independent experiments. Data in **(C, D)** were analyzed using one-way ANOVA followed by Dunnett's multiple-comparisons test, whereas data in **(E)** were analyzed using two-way ANOVA followed by Šídák's multiple-comparisons test [*P* < 0.0001(****)].

**Table 1:** Distribution of spot-assay lysis produced by phage vB_EcoP_P61425 among *E. coli* isolates according to sample source.

Source	No. of tested strains	No. of spot-positive isolates	Lysis rate (%)
Human urine	27	11	40.74%
Chicken liver	23	14	60.87%
Chicken brain	56	38	67.86%
Chicken heart	11	2	18.18%
Sheep lung	6	1	16.67%
Total	123	66	53.66%

Detailed information for all 123 individual *E. coli* isolates, including strain designations, specific origins, O-serotypes, and phage susceptibility profiles, is provided in Supplementary Table S1.

### 3.2 Genomic characterization of vB_EcoP_P61425

Genomic analysis demonstrated that vB_EcoP_P61425 possesses a double-stranded DNA genome of 77933 bp, with a GC content of 42.60%. VIRIDIC analysis of vB_EcoP_P61425 and the 20 related phages selected by BLASTn showed that vB_EcoP_P61425 shared its highest intergenomic similarity of 88.2% with *Escherichia* phage ECBP2 (Figure 2A). This value was below the 95% species-demarcation threshold, supporting the classification of vB_EcoP_P61425 as a distinct species within the genus *Suseptimavirus*. A total of 118 coding sequences were predicted (Figure 2B), and no known lysogeny-associated, antimicrobial-resistance, virulence, or toxin genes were detected. In the global ViPTree proteomic tree, vB_EcoP_P61425 was placed in the “Others” group, comprising phages not assigned to established viral families, and was positioned among phages infecting hosts within the phylum *Pseudomonadota* (Figure 2C). In the local ViPTree proteomic subtree, vB_EcoP_P61425 was positioned most closely to *Escherichia* phage ECBP2, indicating that ECBP2 had the closest genome-wide proteomic relationship to vB_EcoP_P61425 among the included phages (Figure 2D). Comparative genomic analysis further revealed conserved genome organization and regions of protein sequence similarity between vB_EcoP_P61425 and ECBP2 (Figure 2E).

**Figure 2:**
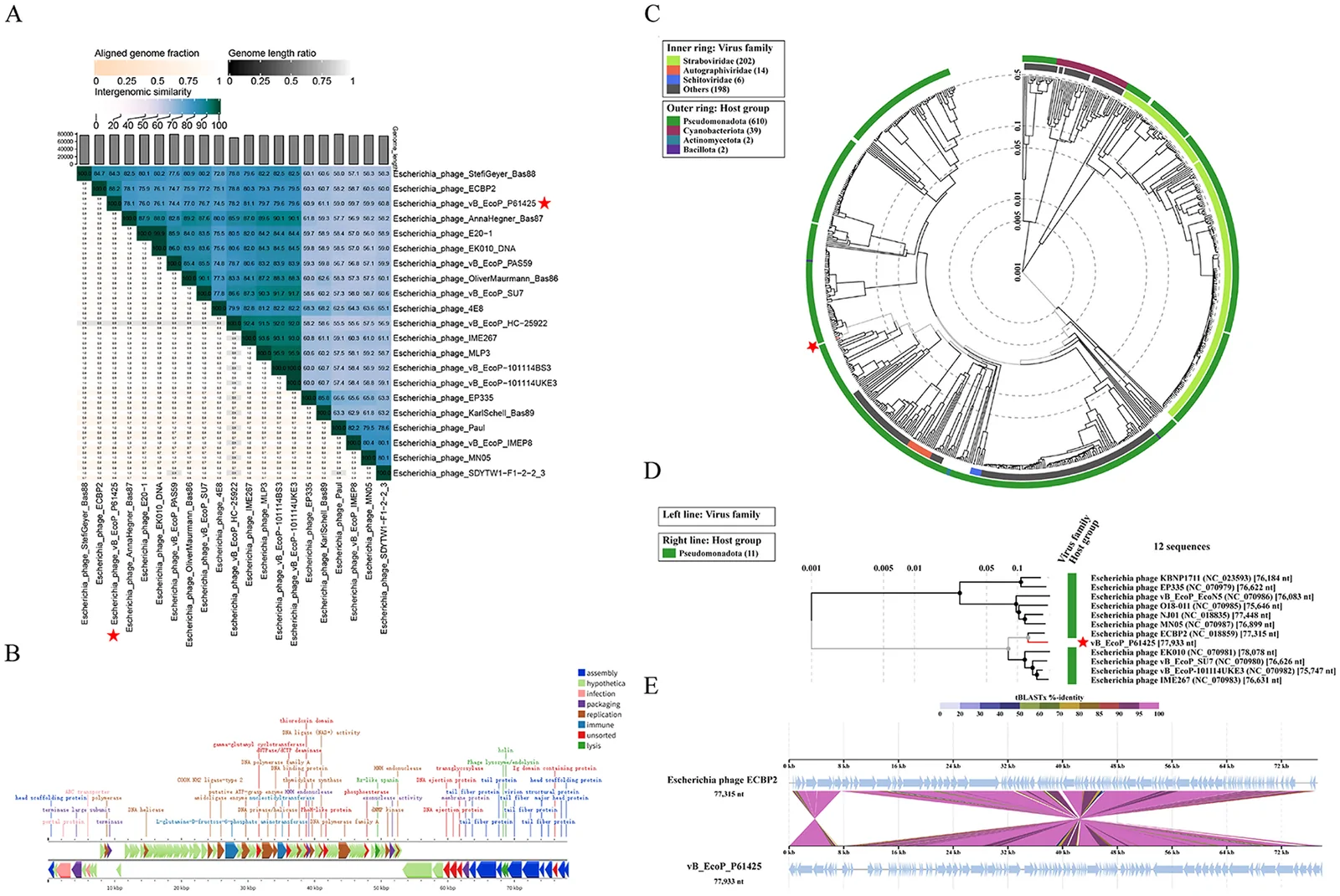
Genomic characteristics and phylogenetic analysis of phage vB_EcoP_P61425. **(A)** Heatmap of pairwise intergenomic similarities calculated using VIRIDIC. **(B)** Linear genome map and functional annotation of predicted coding sequences (CDSs) of phage vB_EcoP_P61425. **(C)** Global viral proteomic tree generated by VipTree analysis based on genome-wide sequence similarities across related viral families. **(D)** Local proteomic subtree generated using ViPTree, comprising vB_EcoP_P61425 and 11 related phages. The subtree was constructed from genome-wide tBLASTx similarity scores using the ViPTree proteomic-tree framework. The left and right annotation bars indicate viral family and host group, respectively. **(E)** Comparative genomic synteny and tBLASTx sequence alignment between phage vB_EcoP_P61425 and *Escherichia* phage ECBP2 visualized using Easyfig. Homologous regions identified by tBLASTx are connected and colored according to their sequence identities. In **(A, C, D)**, vB_EcoP_P61425 is indicated by a red star.

### 3.3 Antibacterial efficacy

All phage treatment groups (MOIs of 0.1, 1, 10) significantly suppressed planktonic EC1649 growth within 6 h compared to the control, with MOI = 0.1 showing the most pronounced inhibition (Figure 3A).

**Figure 3:**
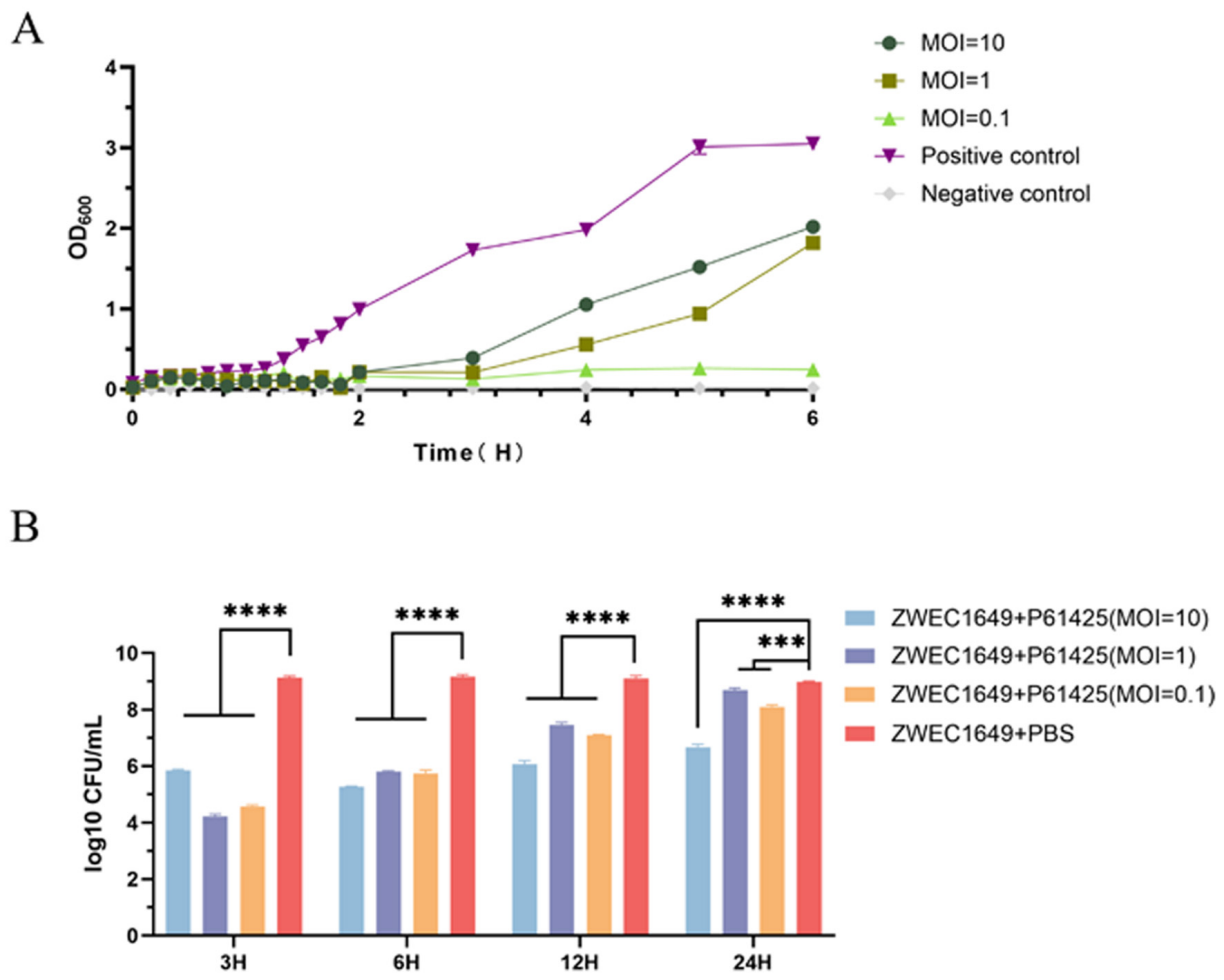
Antibacterial efficacy of phage vB_EcoP_P61425 *in vitro* and in a catheter colonization model. **(A)** Inhibition of planktonic bacterial growth in liquid culture within 6 h. **(B)** Reduction of adherent bacteria in a medical catheter model over 24 h (at 3, 6, 12, and 24 h). Data are presented as the mean ± SD from three independent biological replicates. Data in **(A)** were analyzed using two-way repeated-measures ANOVA, with treatment and time as factors, followed by Dunnett's multiple-comparisons test comparing each phage-treated group with the untreated bacterial control at each time point. Data in **(B)** were analyzed using ordinary two-way ANOVA, with treatment and time as factors, followed by Dunnett's multiple-comparisons test comparing each phage-treated group with the PBS control at each time point [*P* < 0.001(***), *P* < 0.0001(****)].

In the medical catheter model, phage treatment effectively reduced the amount of adherent bacteria. At 3 h and 6 h treatment, viable bacterial counts in the MOI = 0.1 and MOI = 1 groups were slightly lower than those in the MOI = 10 group. In contrast, at 12 h and 24 h, bacterial loads in the MOI = 10 group remained lower than those in the other two groups, indicating a more sustained inhibitory effect (Figure 3B).

### 3.4 Screening and identification of phage-resistant bacteria

Following 24 h co-culture of EC1649 and vB_EcoP_P61425 on LB agar, small white colonies emerged (Figure 4A). Serial passaging yielded three stable mutants (UPR1, UPR2, UPR3) showing complete phage resistance in spot tests (Figure 4B). Sequencing and alignment revealed that UPR1 and UPR2 harbored frameshift mutations in the *wbbL* gene (Figure 4C), whereas UPR3 carried a nonsense mutation in the *waaG* gene (Figure 4D). Morphological observation on blood agar plates showed that, compared with the parental strain EC1649, colonies of UPR1 and UPR2 exhibited a rough edge, whereas UPR3 colonies were markedly smaller and more convex (Figure 4E). Phage adsorption assays demonstrated that the adsorption rates of UPR1, UPR2, and UPR3 were significantly reduced relative to EC1649 (Figure 4F).

**Figure 4:**
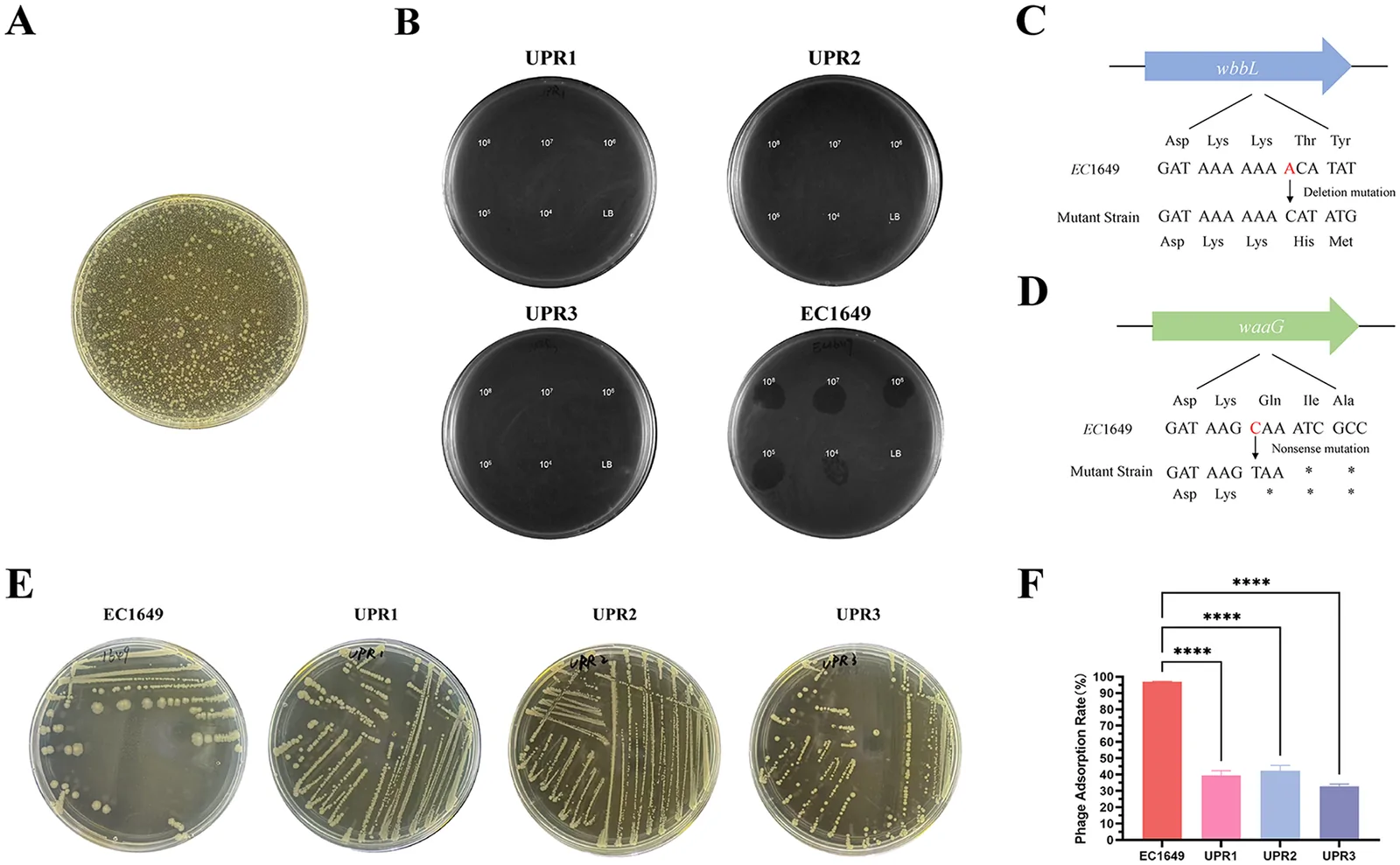
Isolation and characterization of phage vB_EcoP_P61425 resistant mutants. **(A)** Double-layer agar plate used to isolate phage-resistant mutants. **(B)** Spot test assay. **(C)** Schematic representation of the genetic alteration in the *wbbL* gene. **(D)** Schematic representation of the genetic alteration in the *waaG* gene. **(E)** Colony morphology observation. **(F)** Phage adsorption assay. Data are presented as mean ± SD from three independent replicates. Statistical significance relative to EC1649 was determined using one-way ANOVA followed by Dunnett's multiple-comparisons test (**** *P* < 0.0001).

### 3.5 Construction of deletion mutants and verification of phage resistance

To elucidate the roles of *wbbL* and *waaG* in phage resistance, we constructed deletion mutants (EC1649Δ*wbbL* and EC1649Δ*waaG*) and the corresponding complemented strains (EC1649Δ*wbbL*-p*wbbL* and EC1649Δ*waaG*-p*waaG*). Spot assay results revealed that no plaques formed on bacterial lawns of EC1649Δ*wbbL* and EC1649Δ*waaG*, indicating that both mutants were resistant to the phage. In contrast, the complemented strains exhibited plaque formation indistinguishable from that of the wild-type strain, confirming that these genes are directly involved in phage susceptibility (Figure 5A). Phage adsorption assays further demonstrated that adsorption efficiency was markedly reduced from 96.8% in EC1649 to 37.7% and 33.4% in the EC1649Δ*wbbL* and EC1649Δ*waaG*, respectively. The adsorption rates were restored to wild-type levels upon complementation, substantiating the association of these two genes with phage adsorption and subsequent resistance (Figures 5B, C).

**Figure 5:**
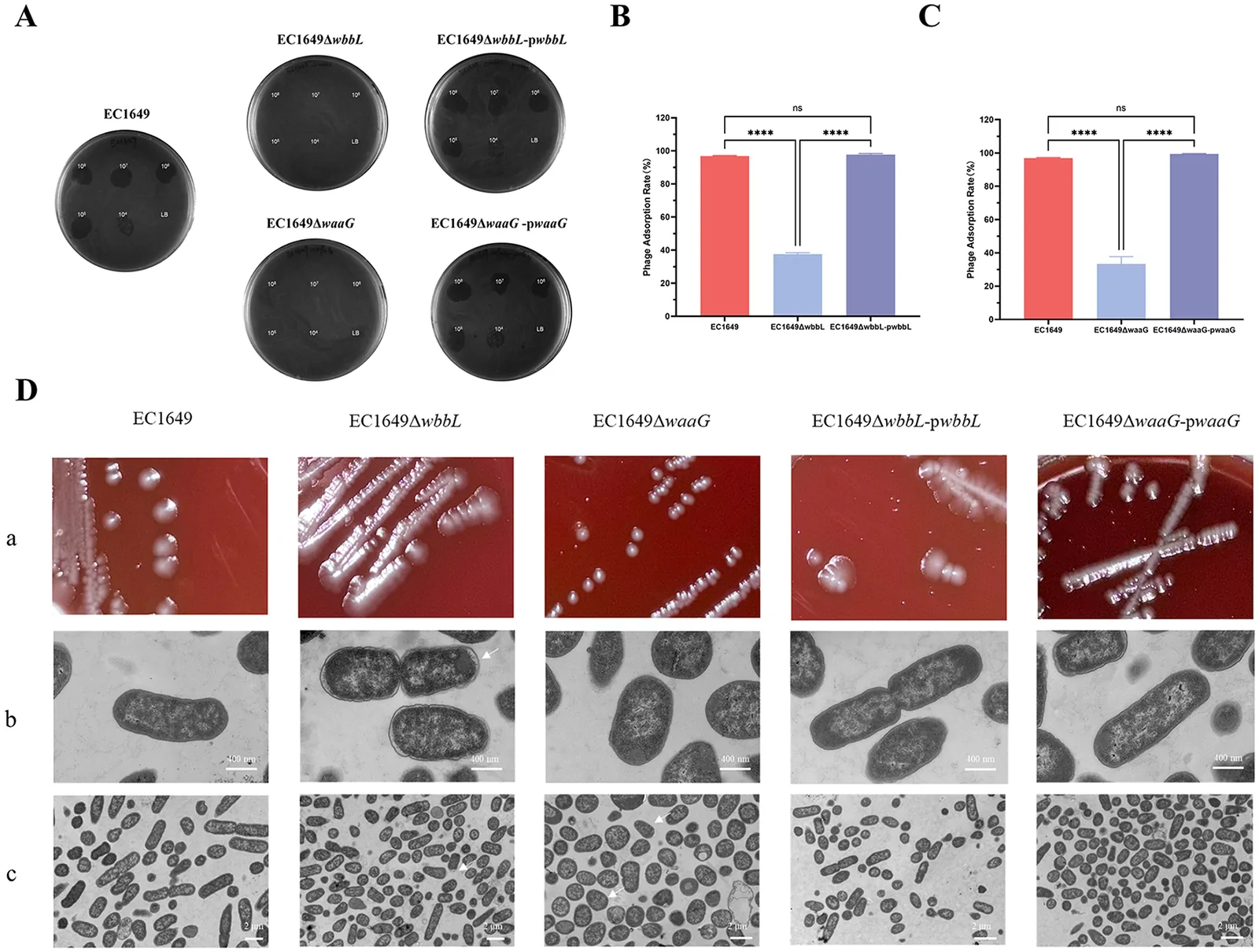
Functional validation of *wbbL* and *waaG* genes in phage resistance. **(A)** Phage susceptibility spot assays. **(B)** Phage adsorption assay of EC1649Δ*wbbL* and EC1649Δ*wbbL*-p*wbbL*. **(C)** Phage adsorption assay of EC1649Δ*waaG* and EC1649Δ*waaG*-p*waaG*. **(D)** Morphological characterization of bacterial colonies and individual cells. (a) Colony morphology. (b) Transmission electron microscopy observations (scale bar = 400 nm). (c) Transmission electron microscopy observations (scale bar = 2 μm). Data in **(B, C)** are presented as the mean ± SD from three independent biological replicates and were analyzed using one-way ANOVA followed by Tukey's multiple-comparisons test (**** *P* < 0.0001; ns, not significant).

Compared with the colonial morphology of EC1649, EC1649Δ*wbbL* displayed a dry, rough-edged appearance, whereas EC1649Δ*waaG* colonies were noticeably smaller and more rounded. Upon complementation with the corresponding wild-type genes, the colony morphology of the mutants reverted to that of the parental strain. Transmission electron microscopy revealed the presence of vesicle-like structures on the outer membrane of EC1649Δ*wbbL* cells. In contrast, EC1649Δ*waaG* cells exhibited a pronounced loss of the typical rod-shaped morphology characteristic of *E. coli*, appearing instead as more rounded forms (Figure 5D).

### 3.6 Cellular adherence capacity

Compared with the EC1649, the three spontaneous phage-resistant mutants (UPR1, UPR2, and UPR3) exhibited a significant decrease in their adherence capacity to HTB-9 cells, with relative adherence rates of 55.11%, 44.17%, and 18.97%, respectively. UPR3 displayed the most severe adherence defect (Figure 6A). To further verify the specific genetic contributions, the adherence capabilities of the deletion and complemented strains were evaluated. The deletion of *wbbL* resulted in a significant reduction in relative adherence to 24.67%, whereas plasmid-borne complementation successfully restored adherence to 82.89% (Figure 6B). Similarly, the *waaG* deletion strain exhibited a reduced relative adherence of 36.11%, which was rescued to 88.47% in the complemented strain (Figure 6C). These results indicate that the acquisition of phage resistance severely attenuates the cellular adherence phenotype of this strain, and that both the *wbbL* and *waaG* genes play crucial roles in maintaining this adherence function.

**Figure 6:**
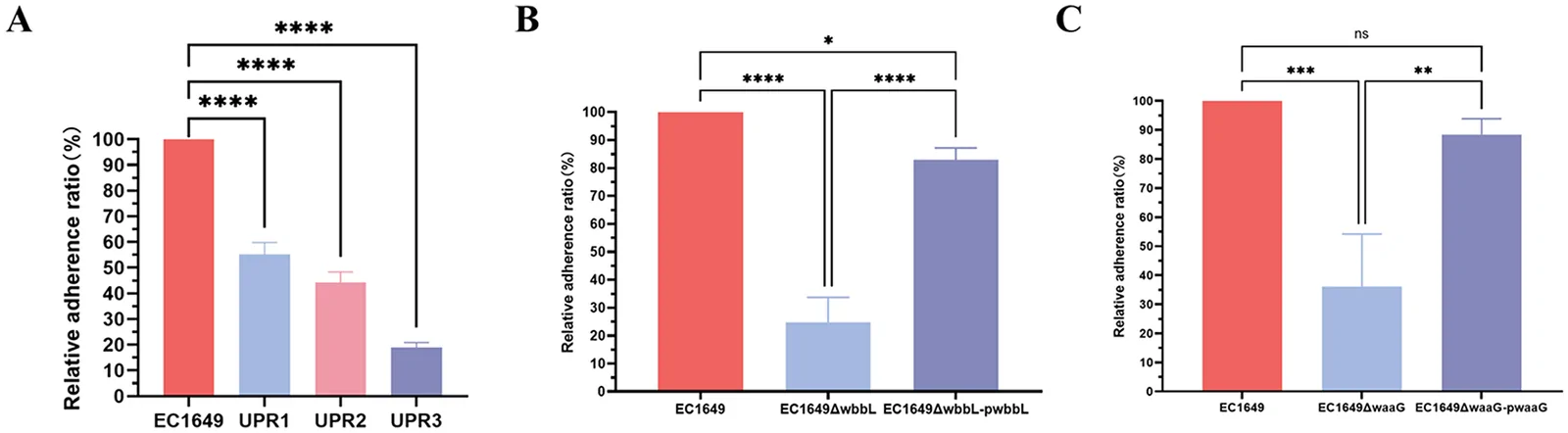
Cellular adherence capacity. **(A)** Relative adherence of spontaneous phage-resistant mutants UPR1, UPR2, and UPR3 to HTB-9 cells compared to the parental strain EC1649. **(B)** Relative adherence of EC1649, EC1649Δ*wbbL* and EC1649Δ*wbbL*-p*wbbL* to HTB-9 cells. **(C)** Relative adherence of EC1649, EC1649Δ*waaG* and EC1649Δ*waaG*-p*waaG* to HTB-9 cells. Data are presented as mean ± SD from three independent biological replicates. Data in **(A)** were analyzed using one-way ANOVA followed by Dunnett's multiple-comparisons test, with EC1649 as the reference group. Data in panels **(B, C)** were analyzed using one-way ANOVA followed by Tukey's multiple-comparisons test [*P* < 0.05(*), *P* < 0.01(**), *P* < 0.001(***), *P* < 0.0001(****); ns, not significant].

### 3.7 Assessment of virulence in a *Galleria mellonella* infection model

The *Galleria mellonella* infection assay demonstrated that *Escherichia coli* EC1649 exhibited potent lethality against larvae, with survival rates decreasing to 30% at 12 h post-infection and stabilizing at 10% from 36 h to the 72 h endpoint, accompanied by severe systemic melanization. Spontaneous phage-resistant mutants UPR1, UPR2 (*wbbL* frameshift mutations) and UPR3 (*waaG* non-sense mutation), as well as the corresponding targeted deletion mutants EC1649Δ*wbbL* and EC1649Δ*waaG* showing 100% larval survival at 72 h, which was identical to the PBS control group. Complementation with either *wbbL* or *waaG* substantially restored bacterial virulence, with larval mortality patterns approaching that caused by EC1649 (Figures 7A–F).

**Figure 7:**
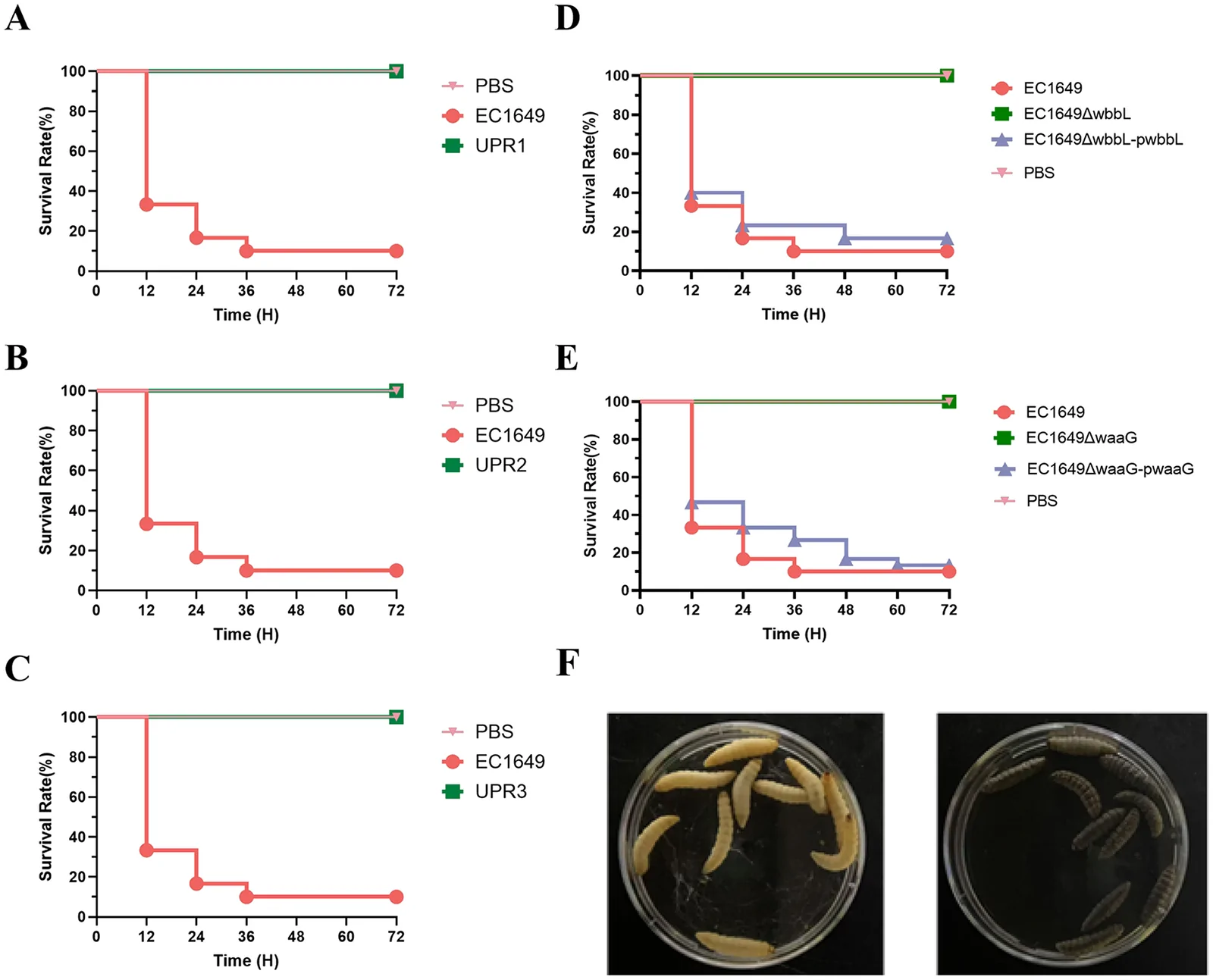
Virulence attenuation associated with phage resistance in the *Galleria mellonella* infection model. **(A)** Survival curve of *G. mellonella* larvae infected with EC1649 and UPR1. **(B)** Survival curve of *G. mellonella* larvae infected with EC1649 and UPR2. **(C)** Survival curve of *G. mellonella* larvae infected with EC1649 and UPR3. **(D)** Survival curve of *G. mellonella* larvae infected with EC1649Δ*wbbL* and EC1649Δ*wbbL*-p*wbbL*. **(E)** Survival curve of *G. mellonella* larvae infected with EC1649Δ*waaG* and EC1649Δ*waaG*-p*waaG*. **(F)** Representative images of surviving larvae without visible melanization **(left)** and dead larvae displaying pronounced melanization **(right)**. Each experiment included 10 larvae per group and was independently repeated three times. Survival curves were generated using the Kaplan–Meier method and compared using the log-rank (Mantel–Cox) test. For multiple pairwise comparisons, *P* values were adjusted using the Holm–Šidák method.

## 4 Discussion

In this study, phage vB_EcoP_P61425 was characterized, and the genetic and phenotypic consequences of phage resistance in ExPEC strain EC1649 were investigated. Among the 123 E. coli isolates tested, 66 showed clearing in the spot assay, whereas 39 reproducibly formed discrete plaques in the double-layer agar assay and yielded quantifiable EOP values. The susceptible isolates included UPEC and APEC strains belonging to clinically relevant serotypes, including O25 and O75 (Han et al., 2025). Genome analysis classified vB_EcoP_P61425 as a distinct species within the genus *Suseptimavirus*, and no known lysogeny-associated, antimicrobial-resistance, virulence, or toxin genes were detected. Its replication characteristics may contribute to the antibacterial activity observed in liquid culture, as rapid phage propagation can enhance bacterial killing under suitable conditions (Abedon et al., 2011). The phage also reduced EC1649 colonization in the catheter model. Although vB_EcoP_P61425 remained active across the tested temperature and pH ranges, these results demonstrate physicochemical stability only under the experimental conditions used. They do not establish adaptation to urine or other *in vivo* environments and are insufficient to support clinical deployment without further evaluation.

Whole-genome resequencing identified frameshift mutations in *wbbL* in UPR1 and UPR2 and a non-sense mutation in *waaG* in UPR3. Targeted deletion of either gene reproduced the resistance phenotype and markedly reduced phage adsorption, whereas complementation restored adsorption and phage susceptibility. These results demonstrate that intact LPS biosynthesis is required for efficient adsorption and infection by vB_EcoP_P61425. LPS contributes to outer-membrane integrity and host–bacterium interactions and comprises lipid A, a core oligosaccharide, and an O-antigen (Klein and Raina, 2019). The *wbbL* gene encodes a rhamnosyltransferase involved in O-antigen repeat-unit synthesis, whereas *waaG* encodes a glycosyltransferase required for LPS outer-core assembly (Rubirés et al., 1997; Yethon et al., 2000). Disruption of these genes is therefore expected to alter surface LPS structures required for phage recognition. The importance of specific LPS structures in phage adsorption has also been demonstrated for other *E. coli* phages and for phages capable of recognizing related LPS structures in different bacterial hosts (Gao et al., 2025; Kaneko et al., 2025). Moreover, *waaG* mutations have previously been associated with resistance to phages T3 and T4 (Perry et al., 2015; Zhong et al., 2020). Nevertheless, the present study did not directly identify the LPS component or epitope bound by vB_EcoP_P61425. The results therefore support an LPS-dependent infection mechanism rather than recognition of a definitively identified LPS structure. Identification of LPS-associated resistance determinants may inform the future selection of complementary phages. Combining phages that depend on different bacterial receptors may reduce cross-resistance and increase the number of mutations required for simultaneous escape (Labrie et al., 2010; Acton et al., 2024; Fujiki et al., 2026). Receptor information may also assist the selection or adjustment of phage combinations when resistance emerges, as illustrated by previous personalized phage applications (Dedrick et al., 2019).

Resistance to vB_EcoP_P61425 was accompanied by alterations in colony morphology, cellular structure, epithelial adhesion, and virulence in the *G. mellonella* model. EC1649Δ*wbbL* exhibited rough colony morphology and abnormal surface structures, whereas EC1649Δ*waaG* showed a more rounded cellular morphology. The spontaneous resistant mutants and the corresponding deletion strains also exhibited reduced adherence to HTB-9 bladder epithelial cells. In the *G. mellonella* model, larvae infected with these strains showed 100% survival at 72 h, compared with 10% survival following EC1649 infection. Complementation of either *wbbL* or *waaG* substantially restored bacterial virulence, reducing larval survival to approximately 15% and producing survival patterns approaching that observed for EC1649. These phenotypes are consistent with the contribution of intact LPS to outer-membrane organization, resistance to host defenses, and bacterial pathogenicity (Burns and Hull, 1998, 1999). Alterations in LPS may also affect host inflammatory responses mediated through Toll-like receptor 4 (Rallabhandi et al., 2008). Nevertheless, the reduced adhesion and larval mortality observed here cannot be attributed to a single mechanism without further structural and functional analyses.

The attenuation observed in the resistant and deletion strains is consistent with previous reports of virulence-associated consequences of phage resistance. LPS-defective ExPEC receptor mutants have shown reduced virulence in mammalian infection models, whereas mutations affecting other surface receptors do not necessarily produce the same outcome (Yan et al., 2025; Gaborieau et al., 2024). Similar associations between phage resistance and virulence attenuation have been reported in *Pseudomonas aeruginosa* and *Klebsiella pneumoniae* (Mi et al., 2023; Yu et al., 2024). These findings are relevant to the concept of phage steering, in which phages targeting structures associated with bacterial pathogenicity may select for resistant variants with attenuated phenotypes (Gurney et al., 2020; Mangalea and Duerkop, 2020). However, the present study did not directly measure bacterial growth rate, generation time, competitive fitness, environmental persistence, or longitudinal evolutionary dynamics. The observed changes should therefore be interpreted as phenotypic and virulence-associated trade-offs rather than evidence of an overall biological fitness cost. Moreover, the experiments did not directly demonstrate treatment-driven phage steering. Further studies should determine the specific LPS structure involved in adsorption, quantify the competitive fitness of resistant mutants, examine resistance trajectories during prolonged phage exposure, and evaluate phage activity, safety, and efficacy in relevant biological fluids and mammalian infection models.

## 5 Conclusion

In summary, we characterized vB_EcoP_P61425, a lytic phage representing a distinct species within the genus *Suseptimavirus*, with activity against multiple ExPEC isolates. Mutational inactivation of the LPS biosynthesis genes *wbbL* and *waaG* in *E. coli* EC1649 markedly reduced phage adsorption and conferred phage resistance. These genetic changes were also associated with altered bacterial morphology, reduced adhesion to HTB-9 bladder epithelial cells, and attenuated virulence in the *Galleria mellonella* infection model. Collectively, these findings demonstrate phenotypic and virulence-associated trade-offs accompanying resistance to vB_EcoP_P61425. Further studies are required to determine whether these trade-offs affect bacterial competitive fitness and can be exploited in mammalian infection models or phage-based therapeutic strategies.

## Data Availability

The complete genome sequence of phage vB_EcoP_P61425 has been deposited in the NCBI GenBank Nucleotide Database (https://www.ncbi.nlm.nih.gov/genbank) under accession number PZ091942.
