# Supplementary material for: Spontaneous wbbL and waaG mutations selected by phage vB_EcoP_P61425 confer resistance and attenuate virulence in ExPEC
Source: Front Microbiol. 2026 Oct 12;17:1953743. doi: 10.3389/fmicb.2026.1953743 (PMC13696500; doi:10.3389/fmicb.2026.1953743)
Supplement: Supplementary file 1 [file Supplementary_File_1.docx]

**Supplementary Material**

**Therapeutic phage vB_EcoP_P61425 induces *wbbL* and *waaG* mutations that attenuate virulence in ExPEC**

**Supplementary Table S1. Strains used for phage host range analysis.**

| Number | Strain | Source | serotype | Spot test | Relative EOP | Classification |
| --- | --- | --- | --- | --- | --- | --- |
| 1 | EC1631 | Human urine | O25 | - | - | - |
| 2 | EC1639 | Human urine | O25 | + | 0.2822 | High |
| 3 | EC1640 | Human urine | O25 | + | 0.1931 | High |
| 4 | EC1645 | Human urine | O25 | + | 0.00495 | Inefficient |
| 5 | EC1647 | Human urine | O25 | - | - | - |
| 6 | EC1651 | Human urine | O25 | + | 0 | None |
| 7 | EC1654 | Human urine | O25 | - | - | - |
| 8 | EC1673 | Chicken brain | O25 | - | - | - |
| 9 | EC1681 | Chicken brain | O25 | - | - | - |
| 10 | EC1682 | Chicken brain | O25 | - | - | - |
| 11 | EC1683 | Chicken heart | O25 | - | - | - |
| 12 | EC1684 | Chicken liver | O25 | + | 0 | None |
| 13 | EC1685 | Chicken heart | O25 | - | - | - |
| 14 | EC1686 | Chicken liver | O25 | + | 1.0248 | High |
| 15 | EC1692 | Chicken brain | O25 | + | 0.9208 | High |
| 16 | EC1693 | Chicken heart | O25 | + | 0.8515 | High |
| 17 | EC1705 | Chicken liver | O25 | + | 0.6931 | High |
| 18 | EC1712 | Chicken liver | O25 | + | 0 | None |
| 19 | EC1713 | Chicken brain | O25 | - | - | - |
| 20 | EC1714 | Chicken liver | O25 | + | 1.0000 | High |
| 21 | EC1717 | Chicken liver | O25 | + | 0.9208 | High |
| 22 | EC1728 | Chicken brain | O25 | + | 0 | None |
| 23 | EC1729 | Chicken brain | O25 | + | 0.0248 | Low |
| 24 | EC1731 | Chicken liver | O25 | + | 0 | None |
| 25 | EC1742 | Chicken brain | O25 | + | 0.00495 | Low |
| 26 | EC1757 | Chicken brain | O25 | + | 0 | None |
| 27 | EC1768 | Chicken brain | O25 | + | 0 | None |
| 28 | EC1665 | Chicken brain | O177 | + | 0 | None |
| 29 | EC1689 | Chicken brain | O177 | - | - | - |
| 30 | EC1694 | Chicken heart | O177 | - | - | - |
| 31 | EC1697 | Chicken brain | O177 | - | - |  |
| 32 | EC1698 | Chicken brain | O177 | - | - | - |
| 33 | EC1702 | Chicken heart | O177 | - | - | - |
| 34 | EC1703 | Chicken brain | O177 | - | - | - |
| 35 | EC1704 | Chicken brain | O177 | - | - | - |
| 36 | EC1706 | Chicken liver | O177 | - | - | - |
| 37 | EC1707 | Chicken liver | O177 | - | - | - |
| 38 | EC1708 | Chicken brain | O177 | - | - | - |
| 39 | EC1709 | Chicken liver | O177 | - | - | - |
| 40 | EC1710 | Chicken heart | O177 | - | - | - |
| 41 | EC1711 | Chicken liver | O177 | - | - | - |
| 42 | EC1721 | Chicken heart | O177 | - | - | - |
| 43 | EC1738 | Chicken brain | O88 | + | 0.0149 | Low |
| 44 | EC1739 | Chicken brain | O88 | + | 0.000990 | Inefficient |
| 45 | EC1740 | Chicken brain | O88 | + | 0 | None |
| 46 | EC1741 | Chicken brain | O88 | + | 0 | None |
| 47 | EC1744 | Chicken brain | O88 | + | 0.000495 | Inefficient |
| 48 | EC1746 | Chicken liver | O88 | + | 0.000495 | Inefficient |
| 49 | EC1747 | Chicken liver | O88 | + | 0.000149 | Inefficient |
| 50 | EC1749 | Chicken brain | O88 | + | 0.000495 | Inefficient |
| 51 | EC1750 | Chicken heart | O88 | + | 0 | None |
| 52 | EC1756 | Chicken brain | O88 | + | 0 | None |
| 53 | EC1758 | Chicken brain | O88 | + | 0.00495 | Inefficient |
| 54 | EC1761 | Chicken brain | O88 | + | 0 | None |
| 55 | EC1628 | Human urine | O75 | + | 0.0426 | Low |
| 56 | EC1635 | Human urine | O75 | - | - | - |
| 57 | EC1637 | Human urine | O75 | - | - | - |
| 58 | EC1638 | Human urine | O75 | + | 0.0104 | Low |
| 59 | EC1641 | Human urine | O75 | - | - | - |
| 60 | EC1643 | Human urine | O75 | + | 0.2624 | Medium |
| 61 | EC1644 | Human urine | O75 | - | - | - |
| 62 | EC1646 | Human urine | O75 | + | 0.2079 | Medium |
| 63 | EC1649 | Human urine | O75 | + | 1.0000 | Reference |
| 64 | EC1726 | Chicken liver | O109 | + | 0 | None |
| 65 | EC1727 | Chicken brain | O109 | + | 00.000990 | Inefficient |
| 66 | EC1745 | Chicken brain | O109 | + | 0.00495 | Low |
| 67 | EC1759 | Chicken brain | O109 | + | 0.000495 | Inefficient |
| 68 | EC1764 | Chicken brain | O109 | + | 0 | None |
| 69 | EC1765 | Chicken brain | O109 | + | 0.000495 | Inefficient |
| 70 | EC1766 | Chicken brain | O109 | + | 0.0139 | Low |
| 71 | EC1767 | Chicken brain | O109 | + | 0 | None |
| 72 | EC1677 | Chicken brain | O16 | + | 0 | None |
| 73 | EC1678 | Chicken brain | O16 | + | 0.0163 | Low |
| 74 | EC1699 | Chicken brain | O16 | + | 0 | None |
| 75 | EC1730 | Chicken brain | O16 | + | 0.000990 | Inefficient |
| 76 | EC1732 | Chicken liver | O16 | + | 0.00302 | Low |
| 77 | EC1733 | Chicken liver | O16 | + | 0.000495 | Inefficient |
| 78 | EC1734 | Chicken brain | O16 | + | 0 | None |
| 79 | EC1642 | Human urine | O18 | - | - | - |
| 80 | EC1650 | Human urine | O18 | - | - | - |
| 81 | EC1653 | Human urine | O18 | - | - | - |
| 82 | EC1743 | Chicken brain | O45 | + | 0 | None |
| 83 | EC1760 | Chicken liver | O45 | + | 0.000990 | Inefficient |
| 84 | EC1763 | Chicken liver | O45 | + | 0.0198 | Low |
| 85 | EC1680 | Chicken brain | O7 | - | - | - |
| 86 | EC1737 | Chicken brain | O7 | + | 0 | None |
| 87 | EC1751 | Chicken brain | O10 | + | 0 | None |
| 88 | EC1755 | Chicken brain | O10 | + | 0.00644 | Low |
| 89 | EC1648 | Human urine | O101 | - | - | - |
| 90 | EC1715 | Chicken liver | O108 | - | - | - |
| 91 | EC1652 | Human urine | O17/O77 | + | 0 | None |
| 92 | EC1672 | Chicken brain | O176 | - | - | - |
| 93 | EC1629 | Human urine | unknown | - | - | - |
| 94 | EC1630 | Human urine | unknown | - | - | - |
| 95 | EC1632 | Human urine | unknown | - | - | - |
| 96 | EC1633 | Human urine | unknown | + | 0 | None |
| 97 | EC1634 | Human urine | unknown | - | - | - |
| 98 | EC1636 | Human urine | unknown | - | - | - |
| 99 | EC1670 | Chicken brain | unknown | - | - | - |
| 100 | EC1675 | Chicken brain | unknown | - | - | - |
| 101 | EC1679 | Chicken brain | unknown | - | - | - |
| 102 | EC1687 | Chicken brain | unknown | - | - | - |
| 103 | EC1688 | Chicken liver | unknown | - | - | - |
| 104 | EC1690 | Chicken liver | unknown | - | - | - |
| 105 | EC1691 | Chicken heart | unknown | - | - | - |
| 106 | EC1695 | Chicken brain | unknown | - | - | - |
| 107 | EC1328 | Sheep lung | unknown | - | - | - |
| 108 | EC1329 | Sheep lung | unknown | - | - | - |
| 109 | EC1330 | Sheep lung | unknown | - | - | - |
| 110 | EC1332 | Sheep lung | unknown | - | - | - |
| 111 | EC1333 | Sheep lung | unknown | - | - | - |
| 112 | EC1334 | Sheep lung | unknown | + | 0.0248 | Low |
| 113 | EC1720 | Chicken liver | unknown | - | - | - |
| 114 | EC1722 | Chicken liver | unknown | - | - | - |
| 115 | EC1723 | Chicken heart | unknown | - | - | - |
| 116 | EC1724 | Chicken brain | unknown | - | - | - |
| 117 | EC1725 | Chicken heart | unknown | - | - | - |
| 118 | EC1735 | Chicken brain | unknown | + | 0 | None |
| 119 | EC1736 | Chicken brain | unknown | + | 0.000495 | Inefficient |
| 120 | EC1752 | Chicken brain | unknown | + | 0 | None |
| 121 | EC1753 | Chicken brain | unknown | + | 0.000990 | Inefficient |
| 122 | EC1754 | Chicken brain | unknown | + | 0.000545 | Inefficient |
| 123 | EC1762 | Chicken brain | unknown | + | 0 | None |

+, lysis positive; −, lysis negative.

**Supplementary Table S2. Primers used in this study.**

| Name | Sequence (5’-3’) | Usage |
| --- | --- | --- |
| wbbL-up-F  *wbbL*-up-R | CGCATGCAAGATCTATCTAGAAGAATGGGATTGATATAACTAGGTTTAAGA  GTATATTAGTGCCTCTGCTCTAATAAAGAAA | Amplification of the upstream homologous arm of *wbbL* |
| *wbbL*-down-F  *wbbL*-down-R | GAGCAGAGGCACTAATATACATATGATAACTCAGTAAGGAAATTTCCC  TCGATACCGTCGACCCTCGAGTCTTCGTTGCATATAACTATTGAATCA | Amplification of the downstream homologous arm of *wbbL* |
| *waaG*-up-F  *waaG*-up-R | CGCATGCAAGATCTATCTAGAACTTGAATCGTTGGTGAAGCAGA  TCGGCGATTTGAACGACGTACCTGTCGTGGAGG | Amplification of the upstream homologous arm of *waaG* |
| *waaG*-down-F  *waaG*-down-R | TACGTCGTTCAAATCGCCGATTTCCAGAA  TCGATACCGTCGACCCTCGAGTCAACCATCCAGACCACCCG | Amplification of the downstream homologous arm of *waaG* |
| *wbbL*-in-F  *wbbL*-in-R | CGGTAATTTCTCATGGTCATT  CGCTGAACCACACTCATCTA | Verification of *wbbL* deletion |
| *wbbL*-out-F  *wbbL*-out-R | ATTGTGGCTCGTTATCATTAAGG  GTTCTGCAACAATAAATCTATGCT | Verification of *wbbL* deletion |
| *waaG*-in-F  *waaG*-in-R | GATCGTTGCTTTTTGTTTATATAA  GGCGATAGAAAAAGCCTTT | Verification of *waaG* deletion |
| *waaG*-out-F  *waaG*-out-R | AGGAAATGACAGATATAACAGTC  CCACCCCATACATAGTATCAAC | Verification of *waaG* deletion |
| PDM4-F  PDM4-R | GCTGACATGGGAATTCCACA  GGTATTTATTCGGCGCAAAGTGC | Confirmation of pDM4 plasmid transfer |
| *waaG*-PGEN-F  *waaG*-PGEN-R | GAAGCTAAACATATGAAGCTTATGATCGTTGCTTTTTGTTTATATAAATA  GGGGCCCGCGGCCGCGGATCCTCAACCATCCAGACCACCCG | Construction of the *waaG* complementation vector |
| *wbbL*-PGEN-F  *wbbL*-PGEN-R | GAAGCTAAACATATGAAGCTTATGATTTATGTTTCGGTAATTTCTCATG  GGGGCCCGCGGCCGCGGATCCTTAAAGCAACTTACTAGAACGCAGC | Construction of the *wbbL* complementation vector |
| pGEN-F  pGEN-R | TCAATAAAGGCACTTGCTCAC  ATCCCAGCAGCAGTTACAAAC | Confirmation of pGEN plasmid transfer |

**Supplementary Figure S1. PCR verification of *wbbL* and *waaG* gene deletion in EC1649.**

(A)wbbL deletion confirmation: External primers (*wbbL* out) shifted from 1037 bp (lane 1) to 663 bp (lane 2), confirming the 374 bp deletion. Internal primers (*wbbL* in) amplified a 311 bp band in wild-type (lane 4) but not in mutant (lane 5).(B) *waaG* deletion confirmation: External primers (*waaG* out) reduced the PCR product from 1901 bp (lane 1) to 1460 bp (lane 2). Internal primers (*waaG* in) yielded a 349 bp amplicon only in wild-type.

**Supplementary Figure S2 PCR-based validation of *wbbL* and *waaG* genetic complementation.**

Lane 1: *wbbL* (1189 bp). Lane 2: *waaG* (1540 bp). Lane 3: empty pGEN vector (415 bp). M: DNA marker. Lane 4: no-template control (no amplification).
